# Dynamics of the *Toxoplasma gondii* inner membrane complex

**DOI:** 10.1242/jcs.147736

**Published:** 2014-08-01

**Authors:** Dinkorma T. Ouologuem, David S. Roos

**Affiliations:** 1 Department of Biology, University of Pennsylvania, Philadelphia, PA 19104, USA; 2 Malaria Research & Training Centre, Department of Epidemiology of Parasitic Diseases, Bamako, BP 1805, Mali

**Keywords:** Apicomplexan parasites, Endodyogeny, Schizogony, Inner Membrane Complex, *Toxoplasma gondii*, *Plasmodium*, FRAP, Photoactivation

## Abstract

Unlike most cells, protozoa in the phylum Apicomplexa divide by a distinctive process in which multiple daughters are assembled within the mother (schizogony or endodyogeny), using scaffolding known as the inner membrane complex (IMC). The IMC underlies the plasma membrane during interphase, but new daughters develop in the cytoplasm, as cytoskeletal filaments associate with flattened membrane cisternae (alveolae), which elongate rapidly to encapsulate subcellular organelles. Newly assembled daughters acquire their plasma membrane as they emerge from the mother, leaving behind vestiges of the maternal cell. Although the maternal plasma membrane remains intact throughout this process, the maternal IMC disappears – is it degraded, or recycled to form the daughter IMC? Exploiting fluorescently tagged IMC markers, we have used live-cell imaging, fluorescence recovery after photobleaching (FRAP) and mEos2 photoactivation to monitor the dynamics of IMC biogenesis and turnover during the replication of *Toxoplasma gondii* tachyzoites. These studies reveal that the formation of the *T. gondii* IMC involves two distinct steps – *de novo* assembly during daughter IMC elongation within the mother cell, followed by recycling of maternal IMC membranes after the emergence of daughters from the mother cell.

## INTRODUCTION

The phylum Apicomplexa comprises thousands of obligate protozoan parasites (Levine, 1970), including clinically significant pathogens, such the *Plasmodium* parasites responsible for malaria (Snow et al., 2005) and *Toxoplasma gondii* – a ubiquitous human pathogen affecting ∼30% of the population worldwide (Pappas et al., 2009). These parasites replicate rapidly in the tissues of susceptible individuals, and pathogenesis is largely a consequence of uncontrolled proliferation (Tenter et al., 2000; Weatherall et al., 2002).

Unlike most cell biological systems where replication has been studied in detail (including bacteria and archaea, as well as animals, plants and fungi), Apicomplexans do not divide by binary fission. Rather, these parasites replicate using a distinctive mechanism in which multiple progeny are assembled within the mother (Hepler et al., 1966; Senaud and Cerná, 1969; Sheffield and Melton, 1968). This unusual process is termed schizogony when daughter nuclei are formed before membrane assembly or endopolygeny when daughter nuclei and membranes develop in parallel (Ferguson et al., 2008). *T. gondii* tachyzoites exhibit a minimal form of endopolygeny, assembling only two daughters within each mother (endodyogeny). These parasites are also readily cultivated *in vitro*, making *Toxoplasma* a useful model system for exploring the biology and mechanism of Apicomplexan parasite replication.

Central to the process of Apicomplexan replication is a membrane–cytoskeletal scaffolding known as the inner membrane complex (IMC) (Hu et al., 2002a; Sheffield and Melton, 1968). Flattened vesicles [cortical alveoli – the major morphological feature unifying the superphylum Alveolata (Adl et al., 2005; Moore et al., 2008)] are positioned immediately beneath the plasma membrane, giving the appearance of a triple membrane (Foussard et al., 1990; Vivier and Petitprez, 1969), which is sometimes called the parasite ‘pellicle’. The outer leaflet of the IMC anchors the actin–myosin motor complex that is required for motility and invasion (Dobrowolski et al., 1997; Frénal et al., 2010; Ménard, 2001), whereas the cytoplasmic side is intimately associated with the subpellicular microtubules and alveolins (intermediate-filament-like proteins) that give the parasite its shape (Mann and Beckers, 2001; Morrissette et al., 1997; Nichols and Chiappino, 1987). Disrupting IMC organization dramatically alters pellicle integrity, cell shape and invasion competence (Khater et al., 2004; Stokkermans et al., 1996; Tremp et al., 2008).

The IMC is also highly dynamic, and its spatial and temporal organization is thought to be crucial for parasite development and replication. At the onset of daughter cell formation, new IMC complexes assemble within the cytoplasm and elongate rapidly, coordinating the segregation of subcellular organelles according to a strict schedule (Nishi et al., 2008). Newly assembled daughters, delimited by the IMC, ultimately emerge from the mother cell, picking up the maternal plasma membrane and sloughing off any residual maternal material (Sheffield and Melton, 1968). Many studies have focused on the cytoskeletal components of the IMC, and several Apicomplexan-specific IMC membrane proteins have been identified (Beck et al., 2010; Bullen et al., 2009; Fung et al., 2012), but our knowledge of alveolar membrane function remains incomplete (Harding and Meissner, 2014). Where does the IMC come from, and how is its assembly and turnover regulated? How does the IMC interact with other organelles during daughter parasite assembly? Exploiting a fluorescently tagged integral membrane protein as a reporter, we have used live-cell imaging and fluorescence recovery after photobleaching (FRAP) to monitor the dynamics of IMC biogenesis and turnover during *Toxoplasma gondii* tachyzoite replication.

## RESULTS

### GAP40 permits the visualization of IMC membrane dynamics during parasite replication

Previous studies on the replication of Apicomplexan parasites have defined the IMC as a valuable morphological marker for tracking the cell cycle, including the assembly of daughter parasites (Hu et al., 2002a; Kono et al., 2012; Nishi et al., 2008). These studies focused on alveolins, such as the IMC1 protein – intermediate-filament-like molecules associated with the inner face of the IMC. In order to understand IMC membrane dynamics, we have employed GAP40, an integral IMC protein with nine predicted transmembrane domains, which is also a component of the glideosome protein complex responsible for parasite motility (Frénal et al., 2010). The Ku80 system (Fox et al., 2009; Huynh and Carruthers, 2009) was used to engineer allelic replacements expressing GAP40–YFP at the endogenous locus in RH strain of *T. gondii* parasites.

**Fig. 1.**
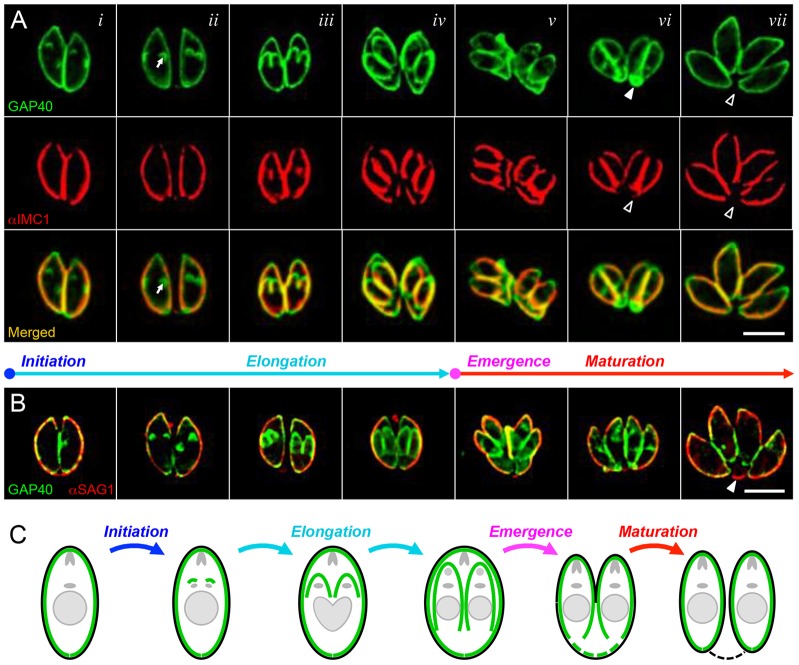
**Stages of the IMC cycle in *T. gondii*.** Colocalization of the integral membrane protein GAP40 (green, A and B) with the cytoskeletal alveolin IMC1 (red, A) and the plasma membrane protein SAG1 (red, B) at various stages throughout the *T. gondii* replicative cycle. Shown are selected images from fixed specimens, approximately corresponding to times *t* = 0 (*i*), 45 min (*ii*), 120 min (*iii*), 180 min (*iv*), 200 min (*v*), 225 min (*vi*) and 360 min (*vii*). Arrows indicate the initiation of GAP40 assembly prior to that of IMC1; arrowheads show that maternal GAP40 disappears from the residual body during maturation. (Note that fixed samples provide better resolution and enable colocalization with SAG1 to be performed; see supplementary material Fig. S1 for time-lapse imaging of GAP40 and IMC1 in living cells.) (C) Cartoon illustrating the relative location of the IMC (green) and the parasite plasma membrane (black), along with the various stages defined in the text – initiation, elongation, emergence and maturation (the color coding of the cartoons is maintained throughout all figures). Scale bars: 5 µm.

GAP40–YFP localizes uniformly throughout the parasite pellicle, including the apical and basal ends, as illustrated in Fig. 1 (see supplementary material Fig. S1 and Movie 1 for time-lapse imaging of living parasites). This contrasts with the localization of IMC1 in several significant ways. First, GAP40 labels the full length of the IMC, whereas IMC1 is excluded from the apical and basal ends (cf. Fig. 1A; supplementary material Fig. S1, merged). Second, GAP40 associates with developing daughter parasites earlier during the replicative cycle than IMC1 (cf. Fig. 1A*i–ii*; supplementary material Fig. S1). Third, maternal GAP40 remains clearly visible throughout the process of daughter parasite emergence from the mother, in contrast to IMC1, which can be difficult to visualize, particularly at late stages (cf. Fig. 1A*vi*; supplementary material Fig. S1).

**Fig. 2.**
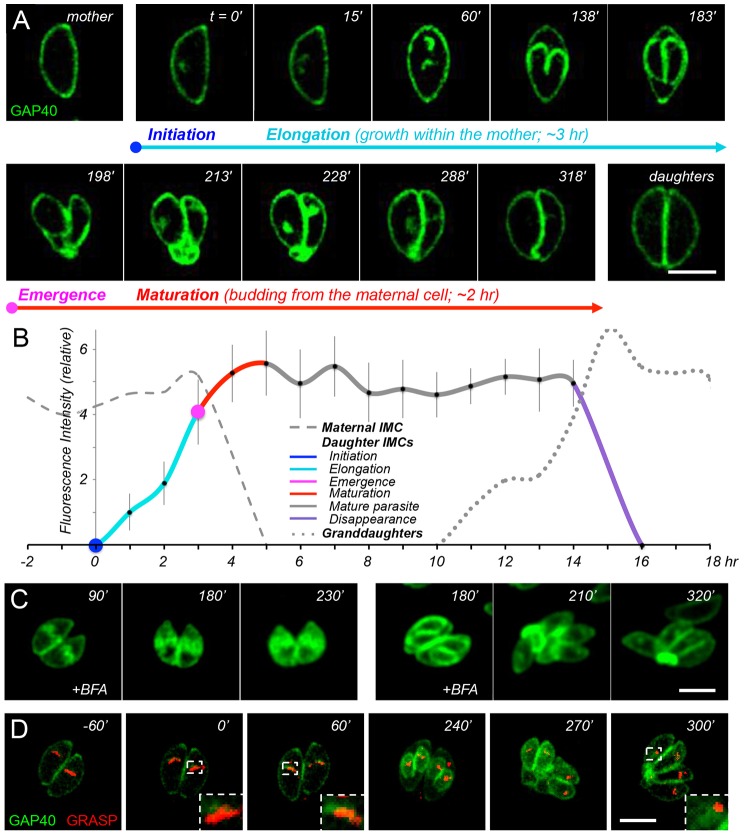
**Time-lapse imaging and quantitative dynamics of GAP40.** (A) Time-lapse imaging of GAP40–YFP-expressing transgenic parasites (C-terminally tagged at the endogenous genomic locus); see supplementary material Movie 1 for additional images. (B) Quantification of GAP40 fluorescence in mother, daughter and grand-daughter parasites (dashed, solid and dotted lines, respectively), determined from 14 sets of time-lapse images aligned according to the estimated time of daughter parasite initiation. The results of a sliding window analysis of 1-h bins are presented as the mean±s.d. (*n* = 6–24 samples; see supplementary material Fig. S2 and Table S1). Color coding indicates IMC initiation (blue), elongation (aqua), emergence (magenta) and maturation (red), as well as interphase parasites (gray) and IMC disappearance (purple). (C) The addition of BFA (5 µg/ml) at 90 min (left) or 180 min (right) after daughter parasite initiation arrests further development of the IMC; see text for discussion. (D) Time-lapse colocalization of GAP40–YFP (green) in transgenic parasites expressing the Golgi marker GRASP–mRFP (red) reveals that assembly initiates close to (but is distinct from) the Golgi (see supplementary material Movie 2 for additional images). The area enclosed by the dashed white line is enlarged in the lower right corner. Scale bars: 5 µm.

We have used the pattern of GAP40 immunofluorescent signal to define several distinct stages in IMC development, as shown by colored text in Figs 1 and 2, and in cartoon form in Fig. 1C. The precise timing of these stages can be determined by live-cell imaging, as shown in Fig. 2A (and the corresponding time-lapse supplementary material Movie 1). Only maternal IMC is visible during interphase (i.e. prior to the initiation of daughter cell assembly, centrosome duplication, Golgi and apicoplast elongation and partitioning).

The first stage of IMC development, initiation of daughter IMC assembly (defined as *t* = 0 throughout this report, and labeled in blue) occurs just apical to the maternal nucleus, adjacent to the microtubule organizing center (MTOC) and Golgi apparatus (Nishi et al., 2008; see also below). As noted above, GAP40 associates with the developing daughter scaffold before the appearance of IMC1. The second stage, daughter elongation (shown in aqua) proceeds basally over the subsequent 3 h, and is defined by growth of the IMC membrane (GAP40) and cytoskeleton (e.g. IMC1). During elongation, maternal organelles are partitioned between daughter parasites, progressively incorporating the centrosome, Golgi, apicoplast and nucleus (with the associated endoplasmic reticulum) (Nishi et al., 2008). The third stage, the emergence of daughter parasites (magenta) occurs by budding from the mother cell ∼180–210 min after initiation, providing daughters with their plasma membrane, as indicated by colocalization with the parasite surface antigen SAG1 (Fig. 1B,C). During the final stage, daughter parasites continue to grow and mature for ∼2 h after emergence from the mother (maturation, red). Daughters remain connected by a narrow cytoplasmic bridge, which is ultimately lost as maternal material is degraded, recycled or left behind in the residual body.

Quantitative analysis of GAP40–YFP fluorescence from multiple parasites over time (Fig. 2B; see also supplementary material Fig. S2 and Table S1 for raw data) demonstrates that this process is highly regular in parasites dividing with normal kinetics. The total fluorescence of developing daughters increases linearly over time, from the earliest stages of initiation (defined as *t* = 0, fluorescence = 0), through elongation and even after emergence from the mother cell, during the final stages of maturation. At ∼5 h after initiation (2 h after emergence), fluorescence intensity reaches a plateau, which is maintained until the beginning of a new replicative cycle.

### Daughter IMCs are assembled *de novo* during elongation

Morphological studies provide useful markers for the parasite cell cycle, but reveal little about the origin of the IMC. As a patchwork of flattened vesicles (Dubremetz and Torpier, 1978; Morrissette et al., 1997; Torpier et al., 1991), it has long been assumed that the IMC must be derived from the Golgi (Agop-Nersesian et al., 2010). Treatment with brefeldin A (BFA) arrests daughter IMC development (Fig. 2C; DeRocher et al., 2005), but GAP40 does not colocalize precisely with the Golgi apparatus (Fig. 2D, especially insets at *t* = 0 and 60 min). GAP40 is more closely associated with a VP1^+^ (vacuolar protein 1) proM2AP^+^ Rab5^+^ organelle known as the endosome-like compartment (ELC) (Tomavo et al., 2013). This organelle is probably comparable to the recycling endosome, or to the pre-vacuolar compartment in plants (Jackson et al., 2013; Robibaro et al., 2002); see below for further discussion.

**Fig. 3.**
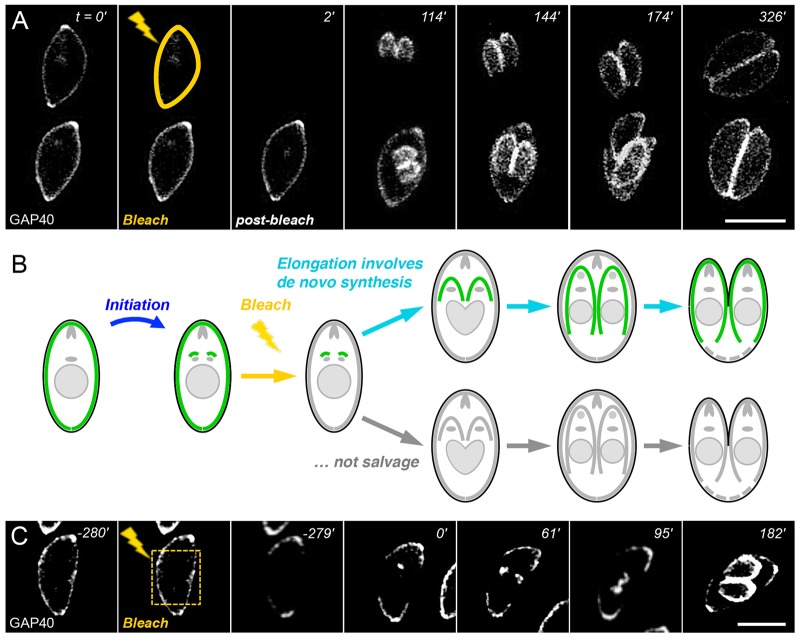
**The daughter IMC is assembled *de novo*.** (A) Laser photobleaching of the maternal IMC (yellow line) has no impact on daughter IMC initiation or elongation, indicating that the daughter IMC is synthesized *de novo* and that newly synthesized GAP40 is not added to the maternal IMC (B). See supplementary material Movie 3 for additional images. (C) Photobleaching only part of the maternal IMC (the area enclosed by the dashed yellow line) also suggests that GAP40 is unable to diffuse in mature parasites. Scale bars: 5 µm.

In order to examine the dynamics of IMC assembly, we tracked parasite replication and GAP40 fluorescence after selectively bleaching either the maternal or daughter IMC. As shown in Fig. 3A (and supplementary material Movie 3), daughter parasite fluorescence appears and elongates with normal kinetics even after the maternal IMC is completely bleached, indicating that the IMC must be synthesized *de novo* (Fig. 3B). No fluorescence recovery was observed in the maternal IMC, indicating that newly synthesized GAP40 is added to daughter IMC scaffolds only.

**Fig. 4.**
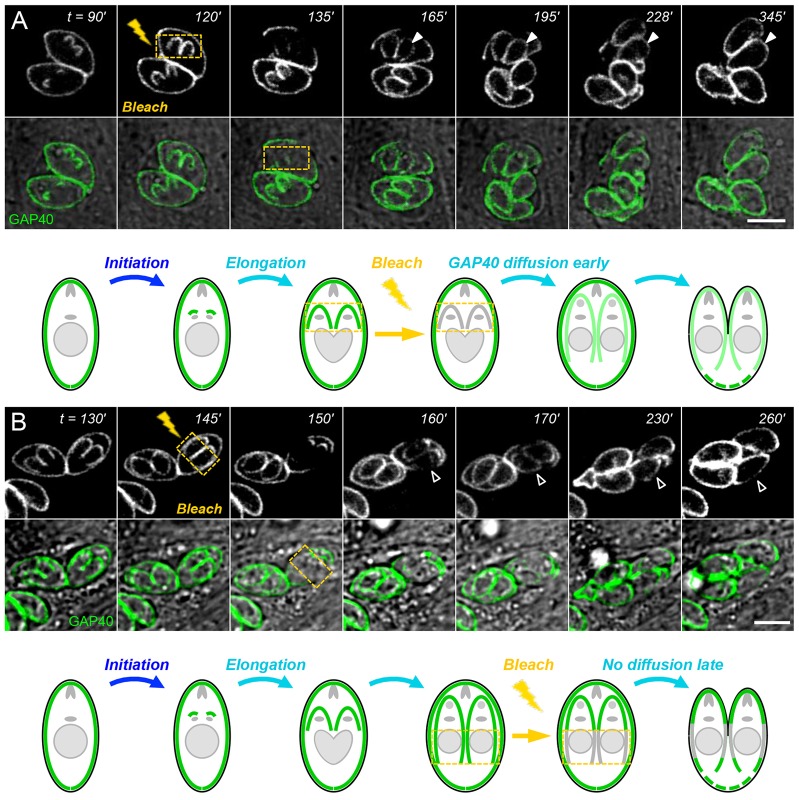
**GAP40 redistribution declines during daughter elongation.** GAP40 fluorescence in daughter parasites recovers rapidly at early stages of elongation (closed arrowheads), owing to a combination of *de novo* synthesis and diffusion or trafficking (A), but fluorescence is unable to fully redistribute when bleaching is applied after ∼150 min post-initiation (B, open arrowheads). Bleached areas are outlined in yellow. Scale bars: 5 µm.

GAP40 is unable to diffuse in mature parasites, as no recovery was observed after bleaching only a portion of the maternal IMC (Fig. 3C). In order to assess the movement of membrane proteins throughout the process of IMC assembly, we bleached the daughter IMC at various stages during elongation and monitored fluorescence recovery, as shown in Fig. 4. Daughter IMCs that are bleached early during elongation (Fig. 4A) recover rapidly, becoming uniformly fluorescent over their entire length, suggesting that newly synthesized IMC is added throughout the entire daughter IMC (or is able to diffuse within the plane of the membrane, or both). Bleaching a portion of the daughter IMC slightly later during the process of elongation results in only partial fluorescence recovery (Fig. 4B). Comparing images from 170 and 160 min shows a slight decline in apical fluorescence and partial fluorescence recovery, but the apical end remains more brightly stained, indicating that GAP40 movement is limited. These data imply that GAP40 is highly mobile at early stages of elongation, but that its mobility declines over time, concomitant with assembly of the membrane–cytoskeletal scaffold.

**Fig. 5.**
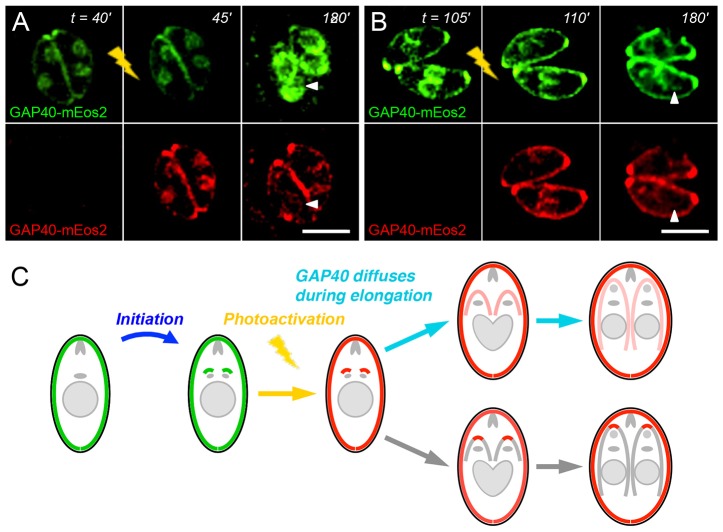
**Photoactivation of GAP40–mEos2 indicates protein movement within the elongating IMC.** Exposure of transiently expressed GAP40–mEos2 to violet light at various times after initiation (A,B) converts a portion of this reporter to a red fluorescent protein. Red fluorescence remains uniform even during elongation (arrowheads), suggesting diffusion within the IMC. (C) Cartoon illustrating the diffusion of red fluorescence (shows red channel only after photoactivation). Scale bars: 5 µm.

Parasites were also transiently transfected with GAP40 fused to mEos2 (Baker et al., 2010; McKinney et al., 2009), a photoconvertible fluorescent reporter that undergoes a conformational change when excited at 405 nm, resulting in a shift from green to red fluorescence. Photoactivation causes a portion of GAP40–mEos2 to fluoresce red (Fig. 5A,B), allowing it to be followed by time-lapse microscopy, analogous to a pulse-chase experiment. During the process of elongation, both red and residual green fluorescence remained essentially constant in maternal parasites, as no additional GAP40 is added to the maternal IMC and maternal GAP40 does not redistribute to daughter parasites (Fig. 3). However, in the developing daughters, whereas green fluorescence increased due to the addition of new material (see above, e.g. Fig. 2), red fluorescence declined, as photoactivated GAP40 disseminated throughout the developing daughter IMC membrane (180 min time-points and Fig. 5C). Total red fluorescence in daughter parasites remained constant but fluorescence intensity per unit of IMC membrane decreased proportionate to the growth of developing daughters.

### Maternal IMC is not left behind in the residual body, but is internalized and incorporated into daughter parasites during maturation

**Fig. 6.**
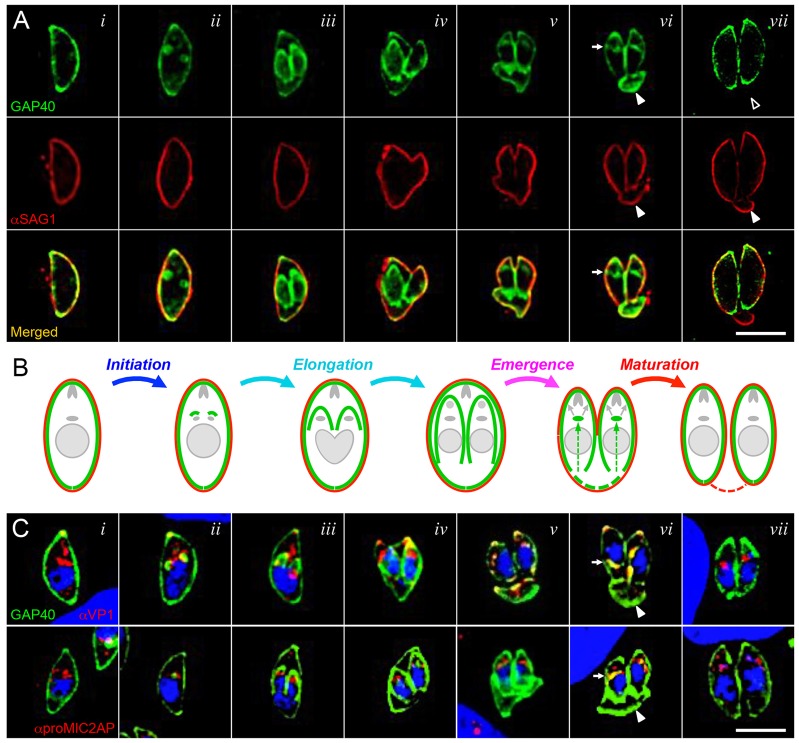
**GAP40 is lost from the residual body and appears in the ELC during daughter parasite emergence and maturation.** (A) Colocalization of the IMC protein GAP40 (green) and plasma membrane protein SAG1 (red) at various stages throughout the *T. gondii* replicative cycle. Selected images from fixed specimens are shown, approximately corresponding to times *t* = 0 (*i*), 30 min (*ii*), 150 min (*iii*), 190 min (*iv*), 200 min (*v*), 225 min (*vi*) and 360 min (*vii*). (B) Cartoon illustrating the relative location of IMC (green) and the parasite plasma membrane (red); arrows in emerging parasites suggest recycling of IMC from the maternal residual body to the daughter IMC. (C) GAP40 (green) traffics to the IMC through the ELC during both elongation (*de novo* synthesis; *ii*) and maturation (salvage of the maternal IMC; *vi*). Arrows indicate the appearance of GAP40 in the Golgi and ELC region of daughter parasites shortly after emergence from the mother (*vi*); arrowheads indicate the residual body. Scale bars: 5 µm.

As daughter parasites emerge, unsegregated maternal material is sloughed off in the ‘residual body’ (Sheffield and Melton, 1968). In contrast to the plasma membrane, maternal GAP40 did not remain in the residual body (Fig. 1C; Fig. 6A, column *vii*). The disappearance of maternal IMC from the residual body coincided with an accumulation of GAP40 in the cytoplasm of emerging and maturing daughter cells [Fig. 6A, column *vi*; also visible in Fig. 1A*vi*,B*vi*; Fig. 2; supplementary material Movie 1 (213–288 min)]. This cytoplasmic accumulation of GAP40 was associated with – but clearly distinct from – the Golgi apparatus (Fig. 2D, 300 min), as previously noted during the early stages of IMC assembly (Fig. 2D, 0–60 min). Colocalization with VP1 (Miranda et al., 2010) and proM2AP (Rabenau et al., 2001) indicated that GAP40 accumulates in the ELC (Robibaro et al., 2001; Tomavo et al., 2013), as shown in Fig. 6C.

**Fig. 7.**
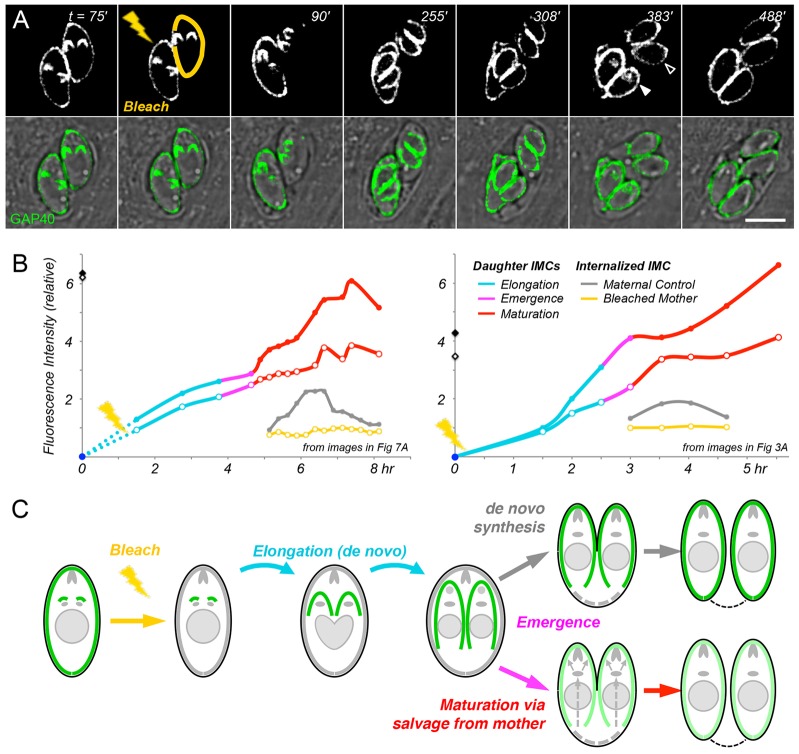
**Maternal IMC is recycled into developing daughters after their emergence.** (A) The entire IMC of one parasite was photobleached (yellow), whereas that of its sister was not (providing an internal control). Time-lapse imaging reveals comparable fluorescence of the daughter IMCs elongating within bleached versus unbleached mothers but, after emergence (308 min), daughters maturing from the unbleached mother are brighter than those from the bleached mother. During the maturation phase, cytoplasmic fluorescence is evident in daughters from the unbleached control parent (filled arrowhead) but not the bleached parent (open arrowhead). (B) Quantification of IMC fluorescence in daughters from unbleached control parasites (solid symbols) versus bleached parasites (open symbols). Panels are based on independent experiments, corresponding to images in Fig. 7A (left; see also supplementary material Movie 4) and Fig. 3A (right; supplementary material Movie 3). Color coding indicates IMC initiation (blue), elongation (aqua), emergence (magenta) and maturation (red). Note that fluorescence is comparable in daughters emerging from bleached versus unbleached mothers through the elongation stage but diverges after emergence, as daughters maturing from unbleached controls remain bright, whereas those from bleached mothers continue to grow without accumulating additional fluorescence. Transient cytoplasmic fluorescence is observed in daughters maturing from unbleached controls (gray) but not bleached mothers (yellow). Filled and open diamonds indicate the fluorescence of parental parasites before bleaching. (C) Cartoon showing *de novo* synthesis of the IMC during elongation and salvage of the maternal IMC during maturation. Scale bar: 5 µm.

To determine whether this late-appearing cytoplasmic pool of GAP40 was actually derived from maternal GAP40, as shown in the cartoon in Fig. 6B, we bleached the maternal IMC in dividing cells and monitored cytoplasmic accumulation of GAP40 after the emergence of daughter parasites (Fig. 7; supplementary material Movie 4). As daughter cells progressed through maturation, a cytoplasmic pool of GAP40 briefly accumulated in the cytoplasm of daughters from control (unbleached) mothers but not in daughters of the bleached mothers (cf. 383 min images in Fig. 7A; supplementary material Movie 4). Moreover, whereas the intensity of daughter parasites was independent of maternal IMC bleaching during elongation (cf. 255 min; Fig. 3), daughter parasites emerging from bleached mothers became progressively dimmer during maturation (cf. 488 min)

Quantification confirmed that the fluorescence of daughter parasites from bleached mothers increased throughout elongation, at an approximately linear rate, comparable to that of daughters from control (unbleached) mothers (aqua lines in Fig. 7B; supplementary material Fig. S3), confirming *de novo* synthesis of the IMC. However, during maturation (red), the fluorescence of daughters from unbleached controls continued to rise (solid symbols), whereas that of daughter cells from bleached mothers slowed (open symbols).

Internal fluorescence arose only in daughters emerging from unbleached mothers (gray versus yellow lines in Fig. 7B). The appearance and disappearance of this material coincided precisely with the disappearance of maternal IMC from the residual body and the cessation of GAP40 addition due to *de novo* synthesis. These observations strongly support the hypothesis that maternal GAP40 is internalized during maturation and recycled into daughter IMCs as illustrated in Fig. 7C.

The replicative cycle of RH strain *T. gondii* is typically ∼7 h, as reported previously (Behnke et al., 2010; Fichera et al., 1995; Nishi et al., 2008), but we observed some variability from specimen to specimen, due to subtle differences in environmental conditions and/or mild phototoxicity during laser photobleaching (parasites that ceased to divide owing to more severe phototoxicity were excluded from analysis). Although atypical, more-slowly replicating parasites provided increased time resolution for the analysis of IMC elongation, maturation and recycling (Fig. 7B, left; supplementary material Movie 4; Fig. S3) and the results obtained from slowly replicating parasites were quantitatively indistinguishable from those of parasites replicating with normal kinetics (Fig. 7B, right; supplementary material Movie 3).

**Fig. 8.**
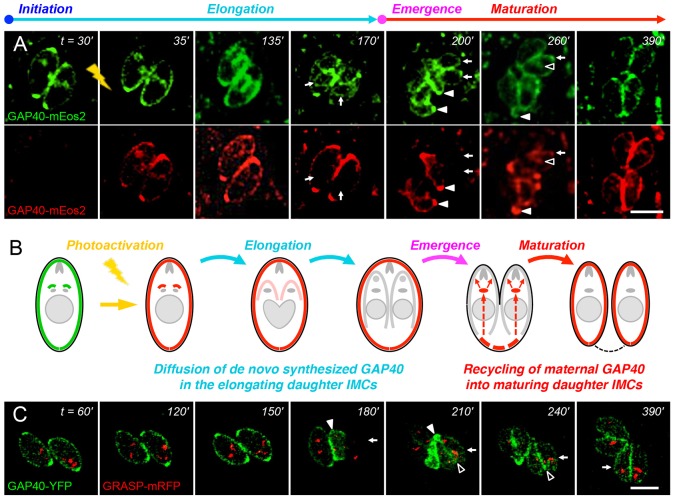
**Photoactivation confirms recycling of maternal GAP40 into daughters during maturation.** (A) Activating GAP40–mEos2 at the onset of daughter formation (lightning) converts a portion of GAP40 from green to red (35 min). The small amount of red IMC in daughter parasites disperses throughout the developing daughter IMC (135 min) and is rapidly diluted by newly synthesized (green) GAP40 (170 min). Maternal IMC is sloughed off in the residual body (closed arrowheads; 200 min), from which it enters into the daughter endomembrane system (open arrowheads; 260 min) *en route* to the daughter IMC (arrows). (B) Cartoon illustrating the diffusion of red fluorescence (shows red channel only after photoactivation). (C) Parasites where only the maternal IMC is visible due to plasmid loss (see text) display IMC in the residual body (closed arrowheads; 180–210 min), recycling through the Golgi and ELC region (open arrowheads; 210 min) and labeling of the daughter IMC (arrows). Scale bars: 5 µm.

The GAP40–mEos2 fusion protein was also exploited to investigate the apparent recycling of maternal IMC during maturation (Fig. 8A,B). As noted previously (Fig. 5), while elongating daughters became increasingly green due to the addition of newly synthesized material, red fluorescence became increasingly difficult to discern because a fixed amount of photoactivated GAP40 dispersed throughout the growing daughter IMC (cf. Fig. 8A, 135 min). By ∼2 h post-activation, green fluorescence was evident in daughter parasites, but the red fluorescence of daughter IMCs had declined to near background levels (arrows in 170 min images). Daughter parasites beginning to emerge from the mother cell at 200 min were clearly green but not red (arrows). At this stage, maternal morphology was lost, as the material that was not incorporated into the developing daughters – including the maternal IMC (red) – was sloughed off in the form of the residual body (solid arrowheads). Over the subsequent 1–2 h (cf. 260 min), maternal IMC was gradually lost from the residual body (solid arrowheads), in parallel with transient internalization of GAP40 into the maturing daughter cytoplasm (open arrowheads) and the appearance of (red, maternal) GAP40 in the mature daughter IMC (arrows). Recycled maternal GAP40 became incorporated throughout the mature daughter parasite IMC (390 min).

Further support for the recycling of maternal IMC into daughter parasites came from transient expression studies, as parasites lost the transfected plasmid over time. When the synthesis of fluorescent GAP40 was fortuitously lost during interphase, the fate of the maternal IMC could be followed in the absence of labeled daughter IMC (Fig. 8C). The maternal IMC was visible in the residual body at the time of emergence (180 min), but later appeared in the daughter IMCs (270 min), trafficking through the Golgi and ELC region (210 min).

## DISCUSSION

Apicomplexan parasites replicate by a highly unusual process, assembling daughter cells within the mother using a membrane–cytoskeletal structure known as the IMC. In addition to providing a scaffold for daughter cell assembly and the maintenance of parasite shape (Khater et al., 2004; Sheffield and Melton, 1968) (Tremp et al., 2008), the mature IMC also serves to anchor molecular complexes that are associated with parasite motility (the glideosome), host cell attachment and invasion (Gaskins et al., 2004; Keeley and Soldati, 2004). By analogy with the alveolae of ciliates and dinoflagellates, the IMC might also play (as yet undefined) roles in storage and/or homeostasis. Most previous studies on Apicomplexan replication have focused on the IMC cytoskeleton (Anderson-White et al., 2011; Anderson-White et al., 2012; Dearnley et al., 2012; Hu et al., 2002a; Hu et al., 2006), but we know little of how the patchwork of flattened vesicles that comprises the IMC membrane is assembled (Torpier et al., 1991).

In order to directly examine IMC membrane assembly, we used the integral IMC membrane protein GAP40 (Frénal et al., 2010) to define four distinct developmental stages – initiation, elongation, emergence and maturation (Figs 1, 2, 6; supplementary material Figs S1–S4). Careful analysis by time-lapse video microscopy, in combination with photobleaching (Figs 3, 4, 7; supplementary material Fig. S3) and photoactivation (Figs 5, 8), revealed that the IMC of *T. gondii* tachyzoites is assembled in two distinct phases – elongation involves *de novo* synthesis (Fig. 3), whereas post-emergence maturation involves salvage and recycling of the maternal IMC (Figs 7, 8). Using modern imaging and analysis techniques, it is possible to quantitatively track the timing of these processes in great detail (Fig. 2B; Fig. 7B; supplementary material Figs S2, S3). The resulting timecourse for tachyzoite development (supplementary material Fig. S4) is remarkably consistent with previous studies (Fichera et al., 1995; Nishi et al., 2008; Behnke et al., 2010), but highlights the entire IMC developmental cycle, including daughter parasite initiation, elongation and emergence (∼3.5 h), followed by maturation (∼2 h), including recycling of the maternal IMC.

Bleaching of the maternal IMC prior to daughter assembly demonstrates that initiation and elongation occur almost entirely through *de novo* synthesis. Although bleached maternal IMC fails to recover, daughter parasites become brightly fluorescent (Fig. 3A) – comparable to the fluorescence of daughters from unbleached mothers (Fig. 7B). This observation supports previous reports that the maternal IMC in dividing *T. gondii* tachyzoites remains intact from the very earliest stages of daughter assembly to the emergence of daughters from the mother cell (Dubey et al., 1998; Hu et al., 2002a; Sheffield and Melton, 1968). Early ultrastructural studies suggested that the daughter IMC might originate as an outgrowth of the maternal IMC (Vivier and Petitprez, 1969), but see below for further discussion.

It is interesting that GAP40 appears to be added to the entire daughter IMC uniformly throughout the process of assembly (Figs 3, 4, 8), suggesting that the IMC grows by expansion in contrast to the processive assembly of subpellicular microtubules (Hu et al., 2002b). FRAP also reveals that GAP40 is able to disperse within the daughter IMC during the first ∼2 h of assembly (Fig. 4A; Fig. 5), but becomes increasingly immobile during elongation (Fig. 4B) and is essentially fixed upon emergence and in mature parasites (Fig. 3C). This contrasts with the cytoskeletal protein IMC1, which is continuously remodeled during IMC assembly (Hu et al., 2002a; Mann et al., 2002). Further studies have failed to distinguish between diffusion versus motor- or vesicle-based trafficking, as the motility of intracellular parasites precludes the analysis of small photobleached spots and protein synthesis inhibitors are toxic to intracellular parasites due to indirect effects on the host cell (data not shown). Nevertheless, it is clear that GAP40 mobility declines with time, presumably as a consequence of association with the microtubule and IMC cytoskeleton, lipid rafts and assembly of the glideosome complex (Johnson et al., 2007; Mann et al., 2002).

New parasites ultimately emerge by budding out of the mother, a process that clothes developing daughter IMCs in the maternal plasma membrane and leaves any remaining maternal material behind in the residual body (Fig. 6). In *Plasmodium* parasites, the residual body includes the polymerized heme residue of hemoglobin digestion (hemozoin). Previous studies on *Toxoplasma* have revealed that the mitochondrion is incorporated into developing daughters very late during development, and that rhoptries and micronemes continue to form during and after emergence. We now report that the daughter IMC membrane also continues to expand after emergence, through salvage of the maternal IMC. Photobleaching clearly demonstrates that daughter IMC growth prior to emergence is independent of the mother, but growth after emergence is almost entirely attributable to salvage of the maternal IMC (Fig. 7). We have termed this newly appreciated phase of the parasite developmental cycle ‘maturation’.

Previous ultrastructural studies (Vivier and Petitprez, 1969) suggested that daughter IMCs might derive from that of the mother, but argued for an early maternal contribution. On the basis of live-cell imaging, we demonstrate that the daughter IMC is indeed derived from salvaged maternal components, but that this recycling occurs only late during the parasite replicative cycle. As noted above, all early IMC synthesis occurs *de novo*, leaving all maternal IMC in the residual body at the point of emergence of daughter cells (Fig. 8A, 200 min; Fig. 8C, 180 min). During maturation, however, maternal IMC disappears from the residual body and becomes incorporated throughout both daughter IMCs (Fig. 8A,C, 390 min). The disruption of both *de novo* IMC assembly (Fig. 2C, left) and IMC salvage (Fig. 2C, right) by BFA treatment, and the association of GAP40 with the ELC (Tomavo et al., 2013) argues that trafficking to the IMC proceeds by the ER–Golgi–ELC pathway. As no vesicles were observed trafficking GAP40 from the residual body to the daughter IMCs, the removal of maternal IMC from the residual body is likely to proceed by fusion with the ER *en route* to the Golgi and ELC (Fig. 6C, Fig. 2D; supplementary material Movie 5).

It remains uncertain what machinery is involved in IMC formation and recycling, in part due to the lack of adequate molecular markers. Dominant-negative Rab11A and Rab11B proteins disrupt the assembly of the glideosome complex and IMC biogenesis, and perturb vesicular trafficking, organellar segregation and cytokinesis, but the causal relationships between these effects is unclear (Agop-Nersesian et al., 2010; Agop-Nersesian et al., 2009). Overexpression of syntaxin 6 (Stx6, which regulates retrograde transport from the ELC to the Golgi) also disrupts vesicular trafficking to the IMC during the late stages of daughter cell assembly (Jackson et al., 2013), suggesting the possible involvement of this protein in maternal IMC recycling. As additional markers are defined, it will be interesting to further explore IMC biogenesis and recycling, and the relationship of these processes to other membrane trafficking pathways (including dense-granule secretion and endocytosis).

*Toxoplasma* tachyzoites divide by endodyogeny, making recycling highly efficient. Other life-cycle stages (and many other Apicomplexan parasites) produce multiple daughters by endopolygeny or schizogony, in which nuclei divide prior to the IMC (Ferguson et al., 2008). Whether the IMC is recycled during the emergence and maturation of these parasites is unknown, but the production of multiple daughters certainly diminishes the potential savings from recycling, as the production of 16 daughters (as is typical during the intraerythrocytic replication of *P. falciparum*, for example) necessitates the production of at least 15 new IMC equivalents. The rapid degradation of the maternal IMC during maturation in *Toxoplasma* might provide useful insights into the rapid IMC degradation observed as *Plasmodium* merozoites differentiate into ring-stage parasites within the infected erythrocyte (Bannister et al., 2000; Hepler et al., 1966). Given the importance of the IMC in parasite structural organization and motility, and the fact that Apicomplexan parasite pathogenesis is a direct consequence of rapid proliferation, better understanding of the IMC might also yield new therapeutic strategies. The availability of good markers for both the cytoskeletal and membrane components of the IMC (Fig. 1), and the high degree of time resolution with which the parasite cell cycle can be defined (Fig. 2B; supplementary material Figs S2, S3) provides useful tools for small-molecule screening.

## MATERIALS AND METHODS

### Cells and parasites

Human foreskin fibroblasts (HFFs) were cultivated at 37°C under a humidified atmosphere containing 5% CO_2_, as previously described (Roos et al., 1995), in a 5∶1 mixture of high glucose Dulbecco's Modified Eagle's Medium (DMEM, Life Technologies, Grand Island, NY) to Medium 199 (Life Technologies), supplemented with 10% newborn calf serum (NBS, Thermo Scientific, Waltham, MA), 50 U/ml penicillin, 50 µg/ml streptomycin and 25 µg/ml gentamicin (Life Technologies). Immediately prior to inoculation of the cells with *T. gondii* tachyzoites, this growth medium was replaced with Minimal Essential Medium (MEM, Life Technologies) supplemented with 2 mM Glutamax (Life Technologies), 1% heat-inactivated fetal bovine serum (FBS, Thermo Scientific) and antibiotics (as above).

### Plasmids

The allelic replacement plasmid *pLic*GAP40YFP-*dhfr*HXGPRT was engineered by PCR amplification of 1358-bp-spanning *Tg*GAP40 (*Tg*ME49_249850; ToxoDB), using the primers shown in supplementary material Table S2, and integration into the *Lic* sequences in *p*YFP.Lic.HXG [kindly provided by Vern Carruthers, University of Michigan (Huynh and Carruthers, 2009)]. Plasmid *pLic*IMC1mCherry-*dhfr*DHFR was engineered similarly, using a 1950-bp fragment from the 3′ end of *Tg*IMC1 (*Tg*ME49_231640) integrated into *p*mCherry.Lic.DHFR. All plasmids were confirmed by restriction digestion and sequencing. After linearization with *Kas*I (for GAP40) or *BsiW*I (for IMC1), 15×10^6^ freshly harvested RHΔKu80ΔHXGPRT strain *T. gondii* tachyzoites (Huynh and Carruthers, 2009) were electroporated with 50 µg of plasmid and were selected in 25 µg/ml mycophenolic acid with 50 µg/ml xanthine (GAP40) or 1 µM pyrimethamine (IMC1) (Roos et al., 1995). Clonal plaques were isolated by limiting dilution and screened by fluorescence microscopy for transgene expression.

Plasmid *ptub*GAP40YFPHA-*sag*CAT was engineered by replacing the ACP sequences in *ptub*ACP-YFP-HA/*sag*CAT*sag* (Nishi et al., 2008) with GAP40 (*Bgl*II–*Avr*II). Plasmid *ptub*GAP40mEos2-*sag*CAT was engineered by replacing the YFP-HA in *ptub*GAP40YFPHA-*sag*CAT with mEos2 amplified as an *Avr*II–*Afl*II fragment from the construct mEos-vinculin [kindly provided by Michael Davidson, Florida State University (Kanchanawong et al., 2010)]. Parasites were transfected with 50 µg of plasmid as above and transient transfectants were examined at ∼18 h post-transfection. All transgenes (YFP, mCherry, mEos2) utilized standard (non-optimized) coding sequences.

### Immunofluorescence microscopy

HFF cells were grown to confluence on 22-mm glass coverslips, infected with *T. gondii* tachyzoites and incubated at 37°C for a further 18–24 h. Coverslips were then fixed for 15–20 min (4% formaldehyde and 0.05% glutaraldehyde in PBS), permeabilized for 15 min (0.25% Triton X-100 in PBS) and blocked for 1 h at room temperature in 3% bovine serum albumin (BSA) fraction V plus 0.25% Triton X-100. After incubation for 1 h with murine monoclonal anti-SAG1 [1∶400 in blocking solution; kindly provided by Lloyd Kasper, Dartmouth College (Mineo et al., 1993)] or anti-IMC1 [1∶2000, kindly provided by Gary Ward, University of Vermont (Mann and Beckers, 2001; Wichroski et al., 2002)] coverslips were washed three times with 0.25% Triton X-100 in PBS and stained for 1 h in Alexa-Fluor-594-conjugated goat anti-mouse-IgG antibody (1∶5000; Life Technologies). For DNA labeling, samples were then incubated for 10 min with 4′,6-diamidino-2-phenylindole dihydrochloride (DAPI, EMD Millipore, Billerica, MA) at a final concentration of 0.5 µg/µl in PBS, washed twice with 0.25% Triton X-100 and once with PBS and mounted on glass slides in Fluoromount-G (Southern Biotech, Birmingham, AL).

Imaging was performed on an Olympus IX70 inverted microscope equipped with a UPlanSApo 100× oil-immersion objective (NA1.4), 300 W xenon arc lamp and a CoolSNAP HQ monochrome cooled-CCD camera. The excitation and emission filters used for DAPI were 360/40 nm and 455/50 nm, respectively; for GFP were 470/40 nm and 520/40 nm, and for mCherry or RFP were 572/35 nm and 632/60 nm. Image stacks were captured using DeltaVision SoftWorx software (Applied Precision, Issaquah, WA) and deconvolved (Figs 1, 6 only) using the constrained iterative algorithm, to minimize the effects of out-of-focus fluorescence. Step size was 0.1 or 0.2 µm and acquisition depth was ∼2–3 µm, satisfying Nyquist sampling. Images were further analyzed using open-source Fiji software (Schindelin et al., 2012) and were imported into PowerPoint for figure preparation.

### Time-lapse microscopy

Confluent HFF cell monolayers were cultivated in 35-mm glass-bottomed dishes (Ibidi, Verona, WI), infected with *T. gondii* tachyzoites at a multiplicity of infection (MOI) of ∼2∶1 [in DMEM lacking Phenol Red (Life Technologies), supplemented with 1% FBS, 1 mM sodium pyruvate, 2 mM glutamine, 100 U/ml penicillin, 100 µg/ml streptomycin and 50 µg/ml gentamicin], and incubated for 12–16 h at 37°C. Prior to imaging, cultures were rinsed with warm PBS lacking divalent cations (to remove extracellular parasites) and incubated in fresh Phenol-Red-free DMEM (as above) supplemented with 10% FBS (to minimize laser phototoxicity) and 25 mM HEPES pH 7. Samples were then transferred to a Chamlide TC stage-top environmental chamber (Live Cell Instruments, Guelph, ON, Canada) that was equipped with a digital temperature, CO_2_ and humidity control unit and equilibrated ∼2 h before data acquisition.

Time-lapse imaging was performed on an Olympus IX-71 spinning-disk confocal microscope equipped with a UPlanSApo 100× oil-immersion objective (NA1.4), CSU-10 scanner (Yokogawa, Newnan, GA), and C9100-13 EMCCD camera (Hamamatsu, Bridgewater, NJ). 5 µm image stacks (26 planes×0.2 µm steps) were acquired by excitation at 488 nm and 561 nm (1% laser power) every 15 or 30 min, using the emission filters ET525/50 for GFP and ET630/75 for mCherry or RFP (Spectral Applied Research). Data was collected using MetaMorph 7.7.4 (Molecular Devices, Downingtown, PA), and processed using MetaMorph and Fiji software. Some images were contrast enhanced for figure presentation.

For quantification of IMC development (Fig. 2), 9–12 time-lapse image stacks (26 planes, as above) were collected over a continuous 9 h session for each of 15 vacuoles in eight fields. At each time-point (for each parasitophorous vacuole) the central image plane was selected from the stack and Fiji software was used to collect perimeter length and total fluorescence (after background subtraction) for all maternal, daughter and/or grand-daughter IMCs, based on manually drawn lines representing all distinctly resolvable IMC structures (supplementary material Table S1a for images and S1b for quantification).

Individual time-lapse series were aligned based on the estimated time of daughter parasite initiation (‘Offset’ column in supplementary material Table S1 and Fig. S2A). Offset values can be reliably estimated to <15 min resolution, as confirmed by the closely coincident timing of daughter parasite emergence at ∼195–210 min, grand-daughter IMC initiation at 600–615 min etc. Multiple vacuoles representing all stages of replication were analyzed, from interphase ‘mother’ parasites shortly after invasion through to the formation of daughter parasites (2 per vacuole) and grand-daughters (4 per vacuole). IMC fluorescence was calculated by sliding-window analysis, pooling samples within hourly windows (e.g. initiation *t* = 0±30 min), and is presented as the mean±s.d. (*n* = 6–24; see supplementary material Table S1 for sample sizes).

### Fluorescence recovery after photobleaching

Photobleaching was performed on the same Olympus IX-71 spinning-disk confocal microscope described above, using a MicroPoint Galvo ablation system (Andor Technology, South Windsor, CT) consisting of a nitrogen-pumped dye laser (Coumarin 440 dye, wavelength 435 nm). Image stacks were processed using MetaMorph 7 (Molecular Devices). Quantification of GAP40–YFP fluorescence (from the individual time-series presented) was carried out using Fiji software as described above, except that cytoplasmic GAP40 fluorescence intensity was collected for emerging and maturing daughter parasites, in addition to the maternal and daughter IMC.

### Photoactivation

For photoactivation studies, *T. gondii* tachyzoites were transiently transfected with *ptub*GAP40mEos2-*sag*CAT, inoculated into HFF cell cultures grown on glass-bottomed dishes as described above, and incubated for 12–16 h at 37°C. Samples were then transferred to an OKOLab stage-top environmental chamber (Warner Instruments, Hamden, CT) that was equipped with a digital temperature and humidity control unit as well as a manual gas controller unit and was equilibrated ∼2 h before data acquisition. Photoactivation was performed using an Olympus IX-81 spinning-disk confocal microscope equipped with an UPlanSApo 100× oil-immersion objective (NA1.4), CSU-10 scanner (Yokogawa) and iXon3 897 EMCCD camera (Andor Technology) and the iLas2 system (Roper Scientific, Paris, France) that employs a 50 mW diode-pumped crystal laser (CrystaLaser model DL405-050-O, wavelength of 405 nm).

Owing to the spontaneous photobleaching properties of mEos2, single images were acquired (no stacks) at selected time-points only using MetaMorph 7.7 (Molecular Devices). Data was collected at 488 and 561 nm (5–25% laser power; emission filters 525/50 nm for GFP and 617/73 nm for TRITC). Some images were contrast enhanced for figure presentation but all quantitative measurements were performed using unprocessed data.

## Supplementary Material

Supplementary Material
