# Supplementary material for: Dynamics of the Toxoplasma gondii inner membrane complex
Source: J Cell Sci. 2014 Aug 1;127(15):3320–30. doi: 10.1242/jcs.147736 (PMC4134349; doi:10.1242/jcs.147736)
Supplement: Supplementary Material [file supp_127_15_3320__index.html]

Dynamics of the Toxoplasma gondii inner membrane complex — Supplementary Material 

# Dynamics of the *Toxoplasma gondii* inner membrane complex

## JCS147736 Supplementary Material

**Files in this Data Supplement:**

- **Supplementary Material**
