# Supplementary material for: Dynamics of the Toxoplasma gondii inner membrane complex
Source: J Cell Sci. 2014 Aug 1;127(15):3320–30. doi: 10.1242/jcs.147736 (PMC4134349; doi:10.1242/jcs.147736)
Supplement: Supplementary Material [file supp_127.15.3320_JCS147736.pdf]

Ouologuem et al, Fig. S1 (top)

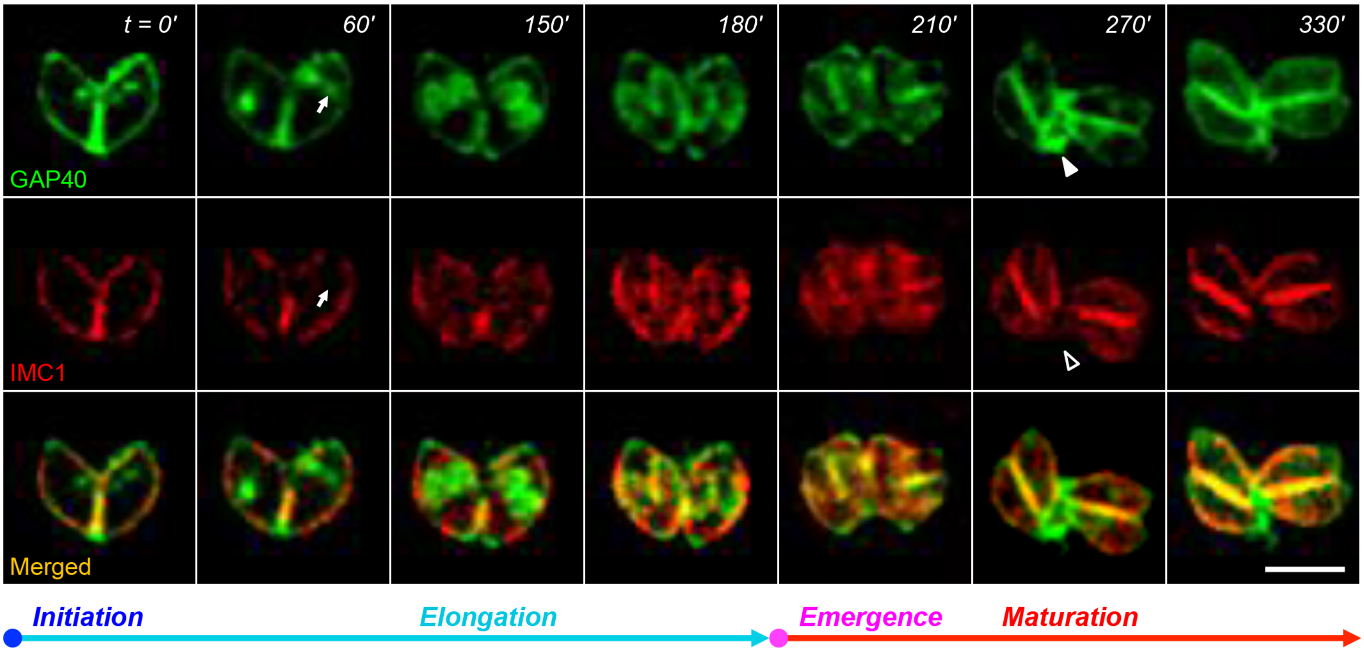

Figure S1. Staging the IMC cycle in living *Toxoplasma gondii* tachyzoites. Time-lapse imaging of *T. gondii* parasites coexpressing GAP40-YFP (transient transfectant) and IMC1-mCherry (endogenously-tagged locus). See Fig 1 legend for further description. Scale bars = 5 µm.

Ouologuem et al, Fig. S2 (top)

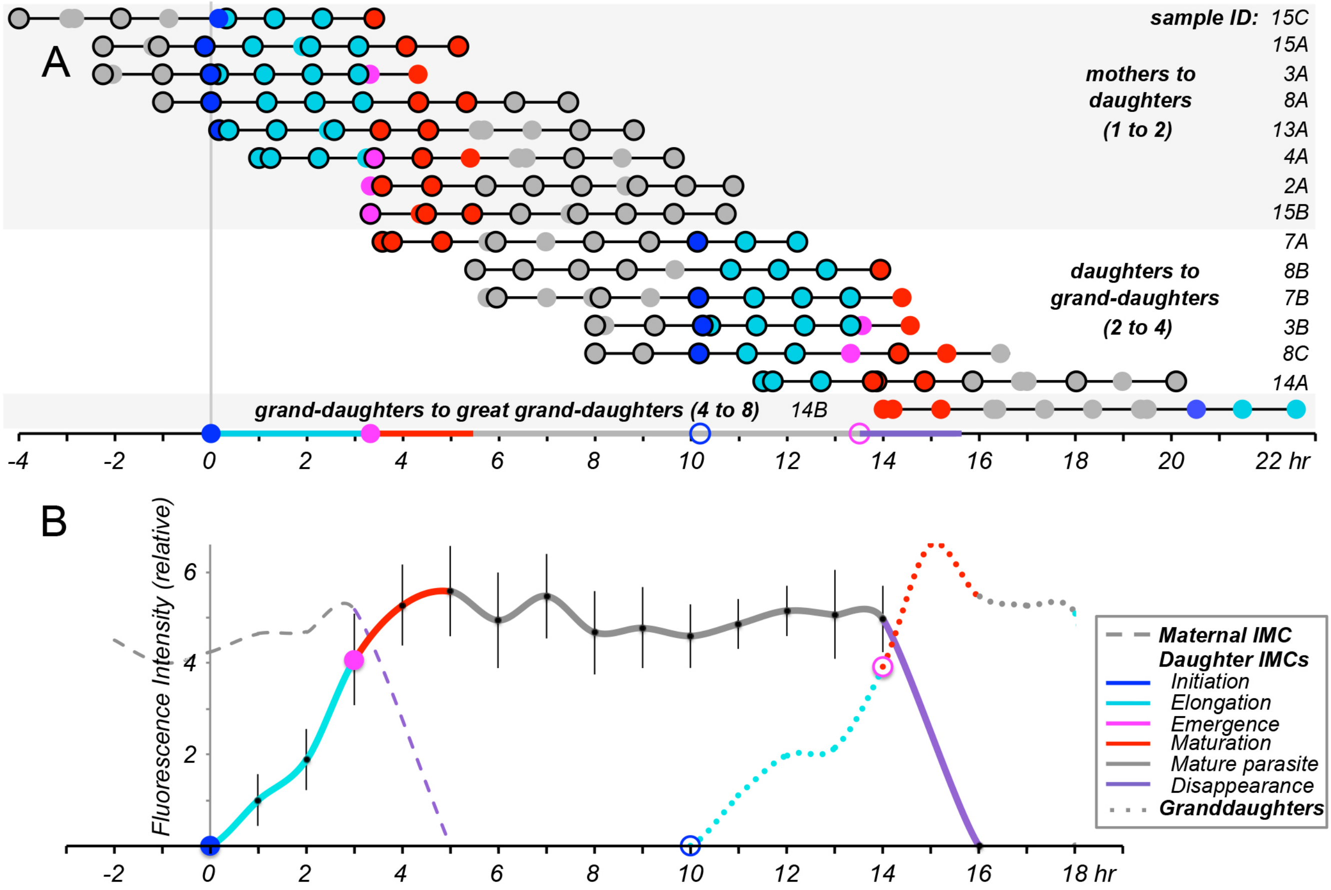

Figure S2. Time-lapse imaging and quantitative dynamics of GAP40. *Panel A,* staging of parasite replication in 9-12 time-lapse images (*dots*) for 15 parasitophorous vacuoles, aligned according to the estimated time of daughter parasite initiation; color coding indicates interphase (*gray*), IMC initiation (*blue*), elongation (*aqua*), emergence (*magenta*), and maturation (*red*). Black circles indicate time points where mother and daughter parasites are readily distinguished, permitting accurate quantification (*Supplementary Table S1 & Fig 2B*). *Panel B (same as Fig 2B),* quantification of GAP40 fluorescence in maternal, daughter, and grand-daughter parasites (*dashed, solid and dotted lines, respectively*), determined from 14 sets of time-lapse images (above); sliding window analysis of 1 hr bins, presented as mean ± s.d. (*n = 6-24 samples; Supplementary Figure S2 and Table S1*). Color coding indicates IMC initiation (*blue*), elongation (*aqua*), emergence (*magenta*), and maturation (*red*); gray indicates interphase parasites, and purple IMC disappearance.

Ouologuem et al, Fig. S3 (top)

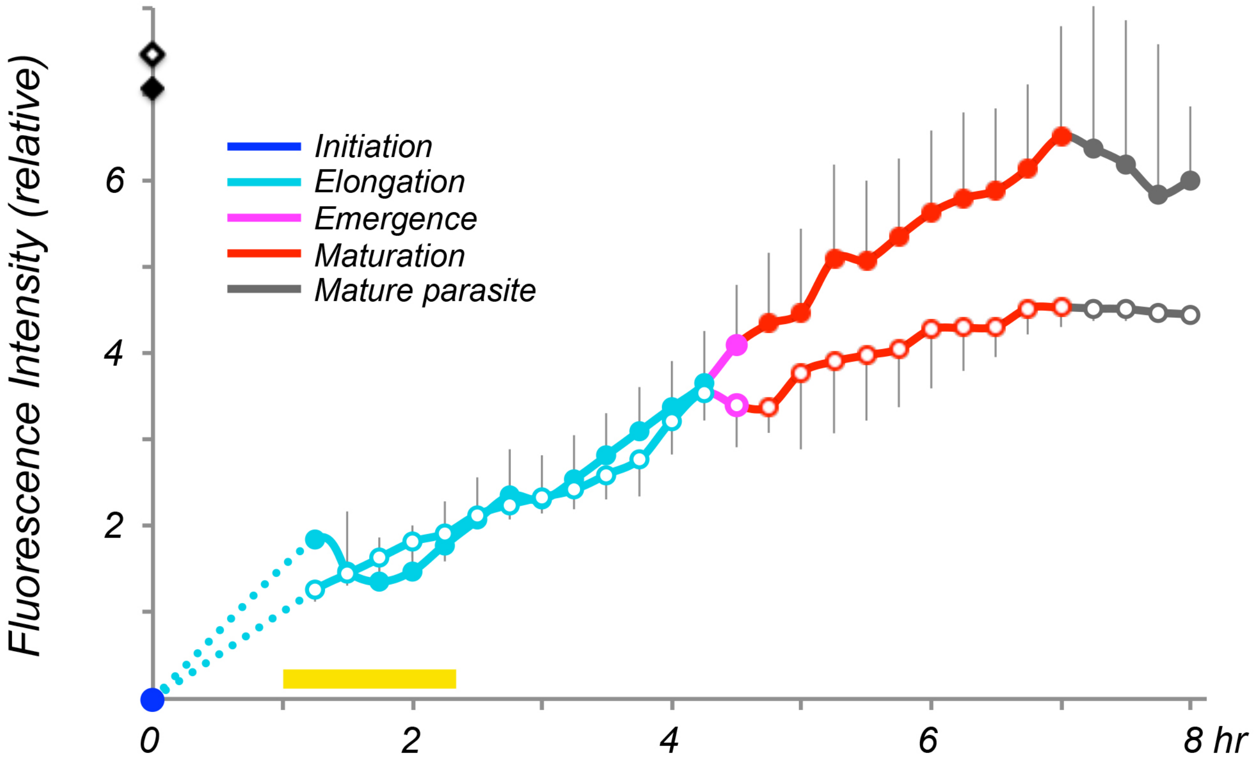

Figure S3. Bleaching of the maternal IMC affects daughter IMC maturation but not elongation. Accumulation of GAP40 in the IMC during the initiation (*blue*) and elonga-tion (*aqua*) of daughter parasites is unaffected by bleaching of the maternal IMC (*open symbols*), but diverges from controls (*closed symbols*) during parasite emergence (*magenta*), indicating that the maternal IMC is recycled into daughter parasites during emergence and maturation (*red*). Curves represent mean ± s.d. for 12 daughter parasites developing within unbleached (control) mothers (*closed symbols*), and 8 daughter parasites developing within mothers bleached at various points within the yellow bar (*open symbols*). Data includes parasites shown in the left-hand panel of Fig 7B. *Filled* and *open diamonds* indicate the fluorescence of parental parasites before bleaching.

Ouologuem et al, Fig. S4 (top)

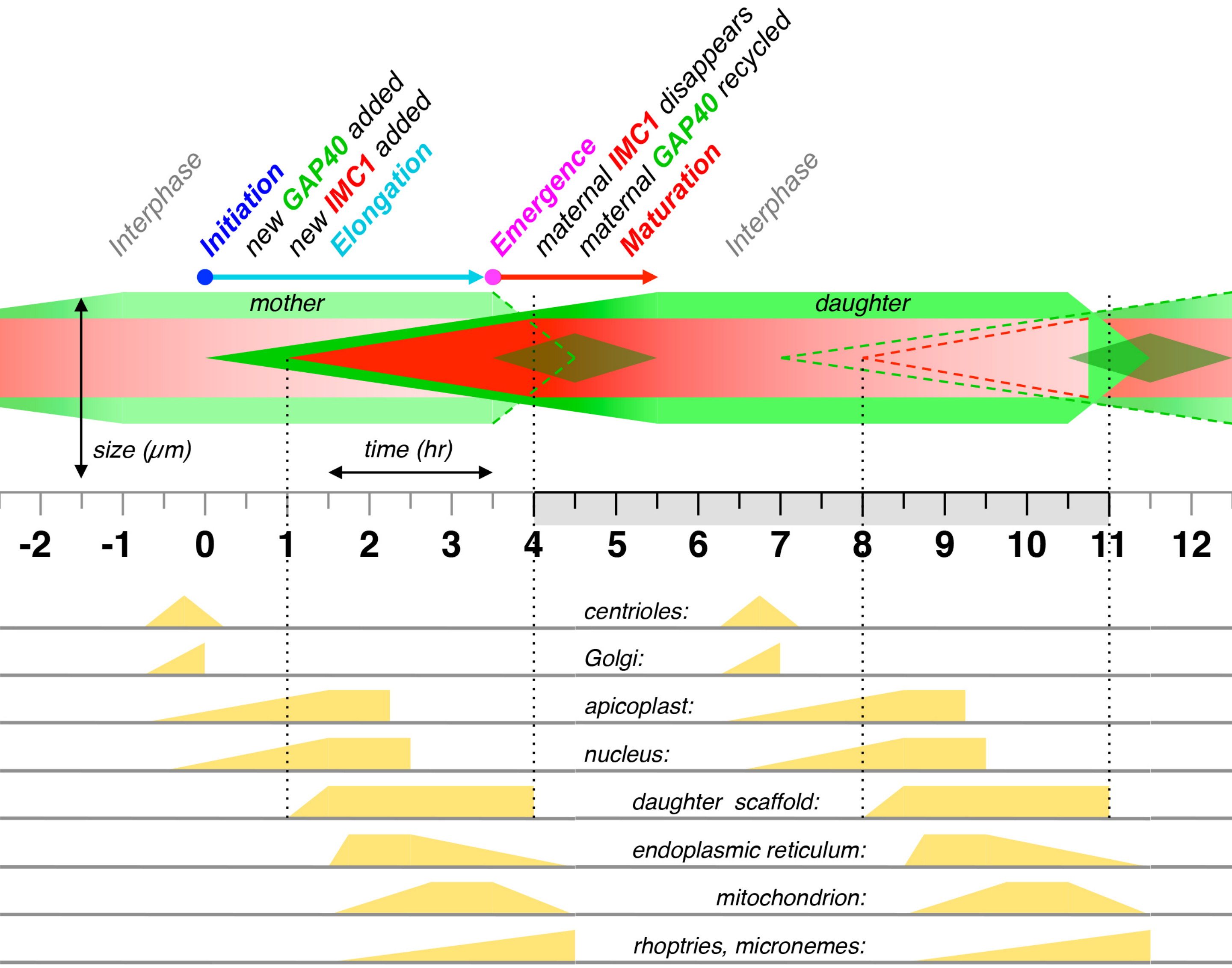

Figure S4. Summary time course of *T. gondii* tachyzoite development. Following the initiation of daughter parasite assembly within the maternal cytoplasm (*blue*), elongation (*aqua*) involves *de novo* synthesis of the IMC membrane (GAP40, *green*) and cytoskele-ton (IMC1, *red*) over the subsequent 3.5 hr. Daughter parasites acquire their plasma membrane as they emerge from the mother (*magenta*), but continue to mature (*red*) over ~2 hr, through the salvage of maternal material (dark shading). Previous studies have focused on organellar segregation during the process of endodyogeny (shaded bar and yellow; *Nishi et al.,* 2008), *i.e.* the 7 hr period between emergence of daughter parasites from the maternal cell, and the emergence of granddaughters. Such analysis fails to fully recognize that complete development of the IMC is an ~11.5 hr process, initiating within the maternal cell, and continuing through dismantling of the daughter cell and salvage of the IMC membrane.

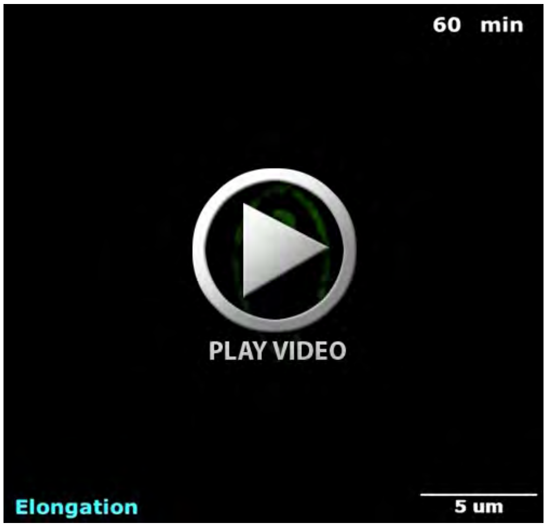

Movie 1. GAP40 Dynamics throughout *T. gondii* replicative cycle. Time-lapse video showing IMC membrane dynamics in GAP40-YFP transgenic parasites (C-terminally tagged at the endogenous genomic locus). Corresponds to Fig 2.

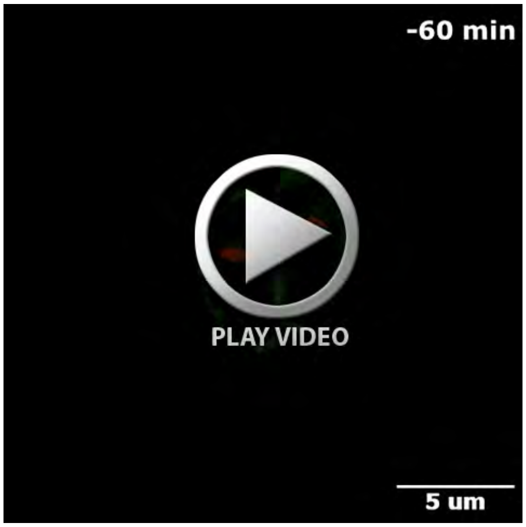

Movie 2. IMC assembly initiates in close proximity to the Golgi apparatus. Time-lapse video of GRASP-mRFP transgenic parasites transiently expressing the IMC marker GAP40-YFP (*green*). Corresponds to Fig S3.

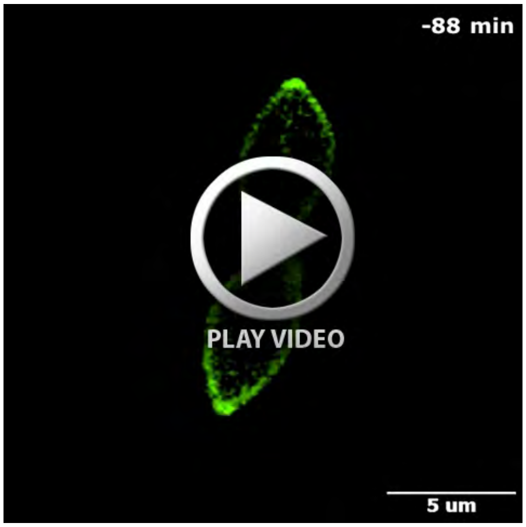

Movie 3. The daughter IMC is assembled *de novo*. Time lapse video showing the *de novo* assembly of daughter IMCs in GAP40-YFP transgenic parasites after photobleaching of the maternal IMC (top; bottom = unbleached control parasites). Corresponds to Fig 3.

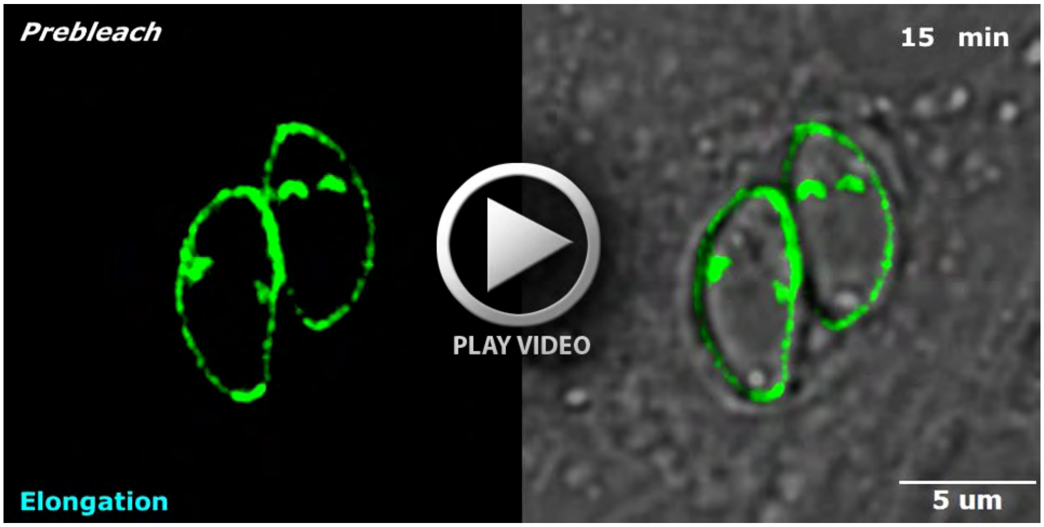

Movie 4. The maternal IMC is recycled into daughter parasites during maturation. Time-lapse series of replicating GAP40-YFP transgenic parasites after photobleaching of the maternal IMC (right; left = unbleached control). Video clearly reveals cytoplasmic accumulation of maternal IMC shortly after emergence from unbleached mothers, and recycling into the maturing IMC of daughters. Cytoplasmic accumulation was not observed in daughters emerging from bleached mothers. Corresponds to Fig 7.

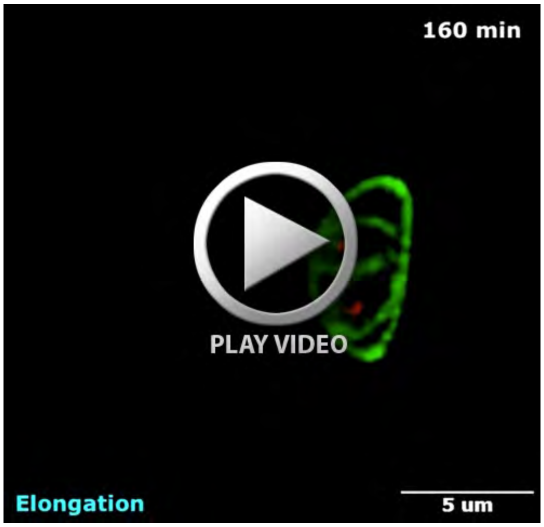

Movie 5. Internalized maternal IMC accumulates transiently in close proximity to the Golgi apparatus during maturation. Time-lapse video of maturing GRASP-mRFP transgenic parasites transiently expressing the IMC marker GAP40-YFP (green). Movie shows cytoplasmic accumulation of maternal IMC near the Golgi apparatus during maturation.

Table S1. Quantification of Gap40 fluorescence in maternal, daughter and grand-daughter parasites. The first page of the Excel spreadsheet displays unenhanced sequential time-lapse images of parasites developing within 15 parasitophorous vacuoles from 8 fields (supplementary material Fig. S2). Vacuoles are marked to indicate parasites used for quantification, as well as these parasites' history (mothers; daughters a or b; granddaughters a1, a2, b1 or b2), and the stage of replication, using the same blue → aqua → magenta → red color scheme for initiation, elongation, emergence and maturation applied throughout this manuscript. Page 2 presents quantitative fluorescence levels, sequentially aligned on the horizontal axis according to the inferred time since initiation (see supplementary material Fig. S2). Results are summed at the bottom of the table, using a sliding window analysis as described above, and presented in graphical form as Fig. 2B.

Download Table S1

Table S2: List of Primers used in this study*

F-Lic-GAP40	5'-*TACTTCCAATCCAATTTAATGC*AGCGTTCGCACAGTTCCTCTGC-3'
R-Lic-GAP40	5’-*TCCTCCACTTCCAATTTTAGC*GCTCGAATGGGCTTCGTCGTCAC-3'
F-Lic-IMC1	5’-*TACTTCCAATCCAATTTAATCG*TTGAGAAGGTAAGACAGGTGGCGC-3'
R-Lic-IMC1	5’-*TCCTCCACTTCCAATTTTAGC*GCACTGGCATCGGCACACACCATCAC-3'
F-*Bgl*II-GAP40	5’-*AGATCT*ATGTCGACTCTTCAGGACATTCGC-3'
R-GAP40-*Avr*II	5’-*CCTAGG*GCTCGAATGGGCTTCGTCGTCACAA-3'
F-*Avr*II-mEos2	5’-*CCTAGG*ATGAGTGCGATTAAGCCAGACATG-3'
R-mEos2-*Afl*II	5’-*CTTAAG*TTATCGTCTGGCATTGTCAGGCAATCC-3'

* Lic sequences and restriction sites shown in italics; *T. gondii* genomic sequence are underlined.
